# Four Weeks of Probiotic Supplementation Alters the Metabolic Perturbations Induced by Marathon Running: Insight from Metabolomics

**DOI:** 10.3390/metabo11080535

**Published:** 2021-08-11

**Authors:** Jamie N. Pugh, Marie M. Phelan, Eva Caamaño-Gutiérrez, S. Andy Sparks, James P. Morton, Graeme L. Close, Daniel J. Owens

**Affiliations:** 1 Research Institute for Sport and Exercise Science, Liverpool John Moores University, Liverpool L3 3AF, UK; J.Pugh@ljmu.ac.uk (J.N.P.); J.P.Morton@ljmu.ac.uk (J.P.M.); G.L.Close@ljmu.ac.uk (G.L.C.); 2 NMR Metabolomics Shared Research Facility, Technology Directorate, University of Liverpool, Crown Street, Liverpool L69 7ZB, UK; Marie.Phelan@liverpool.ac.uk; 3 Computational Biology Facility, Technology Directorate, University of Liverpool, Crown Street, Liverpool L69 7ZB, UK; Eva.Caamano-Gutierrez@liverpool.ac.uk; 4 Sport Nutrition and Performance Research Group, Department of Sport and Physical Activity, Edge Hill University, Ormskirk L39 4QP, UK; sparksa@edgehill.ac.uk

**Keywords:** metabolomics, marathon, exercise, probiotic, metabolism

## Abstract

Few data are available that describe how probiotics influence systemic metabolism during endurance exercise. Metabolomic profiling of endurance athletes will elucidate mechanisms by which probiotics may confer benefits to the athlete. In this study, twenty-four runners (20 male, 4 female) were block randomised into two groups using a double-blind matched-pairs design according to their most recent Marathon performance. Runners were assigned to 28-days of supplementation with a multi-strain probiotic (PRO) or a placebo (PLB). Following 28-days of supplementation, runners performed a competitive track Marathon race. Venous blood samples and muscle biopsies (vastus lateralis) were collected on the morning of the race and immediately post-race. Samples were subsequently analysed by untargeted ^1^H-NMR metabolomics. Principal component analysis (PCA) identified a greater difference in the post-Marathon serum metabolome in the PLB group vs. PRO. Univariate tests identified 17 non-overlapped metabolites in PLB, whereas only seven were identified in PRO. By building a PLS-DA model of two components, we revealed combinations of metabolites able to discriminate between PLB and PRO post-Marathon. PCA of muscle biopsies demonstrated no discernible difference post-Marathon between treatment groups. In conclusion, 28-days of probiotic supplementation alters the metabolic perturbations induced by a Marathon. Such findings may be related to maintaining the integrity of the gut during endurance exercise.

## 1. Introduction

During strenuous exercise, the gastrointestinal (GI) tract faces a number of stressors, and one of the consequences, most commonly seen in endurance runners, is an increase in symptoms such as bloating, abdominal cramping, diarrhoea, nausea, and vomiting [1,2]. The aetiology of GI distress during endurance exercise is, in part, related to splanchnic hypoxia, oxidative stress, hyperthermia, mechanical stress related to exercise, and malabsorption of carbohydrates (CHO) consumed before and during exercise [3,4]. Reduced CHO absorption due to GI distress poses a particular problem for endurance athletes, as CHO availability during endurance exercise lasting > 60 min may be a limiting factor for performance. As liver and muscle glycogen stores are limited, oral ingestion of CHO before and during exercise improves performance and delays fatigue in cycling and running [5,6]. Due to the apparent importance of the microbiome on mammalian metabolism, GI morphology and integrity, and overall health [7], there has been an interest in probiotic supplementation, with the aim of altering the existing GI environment. Such supplementation has been associated with a number of benefits for different aspects of human health and, more recently, there has been significant interest in how probiotic supplementation could positively impact the health and performance of athletic populations, including endurance athletes [8,9,10].

Few studies have investigated whether probiotic supplements can reduce GI symptoms and help maintain endurance exercise performance. A reduction in the duration of GI symptoms was noted in a group of recreational runners two weeks after a Marathon following a single strain probiotic supplementation, although only severe symptoms (diarrhoea, vomiting, and stomach-ache) were recorded, and no differences were found during the period of supplementation [11]. The severity of GI symptoms during training in novice triathletes was also reduced when supplementing with a multi-strain probiotic [12]. Recently, we have shown in a randomised controlled trial of twenty-four recreational runners, that supplementation with a multi-strain probiotic for 28 days prior to a Marathon race reduces the incidence and severity of GI symptoms when compared with a placebo control, which was also associated with better maintenance of running pace in the latter stages of the Marathon [10]. We were unable to associate the improvement in GI symptoms with any one of the biochemical variables assessed in our study, and, moreover, markers of GI damage were no different between treatment groups. Our previous findings suggest that the potential positive effects of probiotics may be in the improved absorption of carbohydrates across the GI tract. However, the precise mechanisms and interactive effects by which probiotics may positively regulate aerobic exercise performance remain to be determined.

Metabolomic profiling has demonstrated that the microbiome has a considerable influence on the mammalian blood metabolome [13], and, therefore, metabolomic profiling of Marathoners ingesting a multi-strain probiotic has the potential to reveal new insights into how probiotics influence systemic metabolism and endurance exercise performance. In a recent publication, untargeted metabolite profiling was performed on the serum of 31 runners prior to and following a Marathon to identify metabolites that vary most due to the physiological stress of this type of endurance exercise [14]. These findings revealed that a Marathon induces a substantial strain on bioenergetic pathways, potentially causing extensive protein degradation, oxidative stress, and inhibition of anabolic pathways. The findings not only highlight the complexity of the physiological responses to Marathon running but also the utility of untargeted metabolomics for better characterising the response to exercise.

The aim of the current study was to generate new hypotheses and subsequently better characterise the mechanisms by which probiotic supplementation may incur beneficial physiological effects during Marathon running. To achieve this aim, samples derived from our aforementioned track Marathon study [10] were analysed by untargeted metabolomics before and after a Marathon race. 

## 2. Results

**Figure 1:**
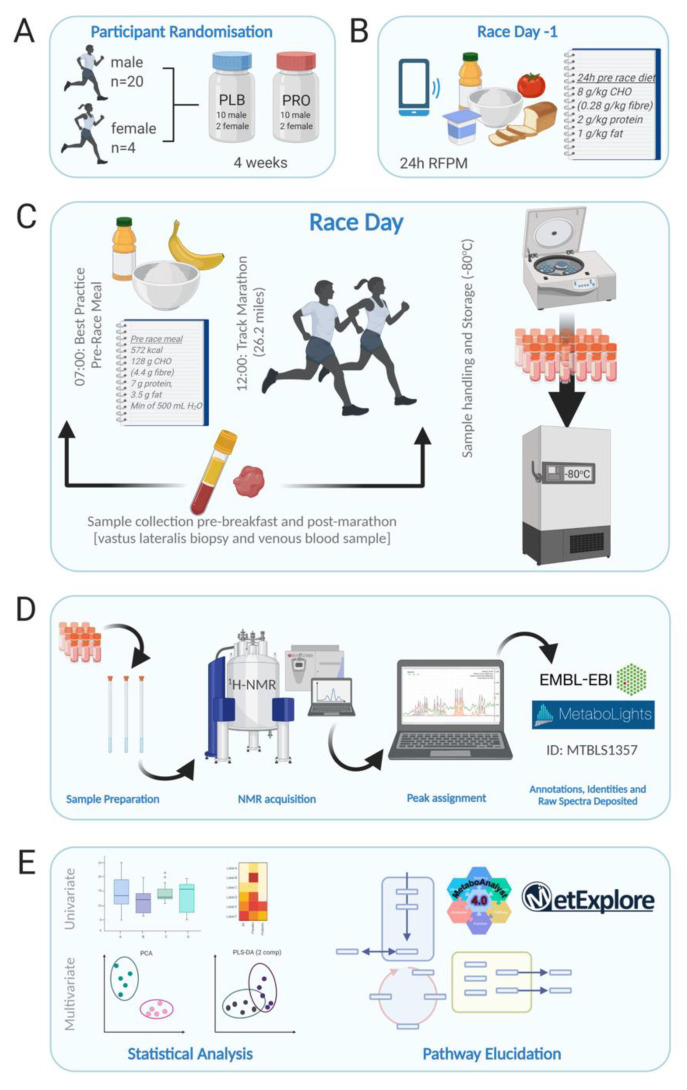
Schematic overview of the study design. (**A**) Participant randomisation (*n* = 24; 20 males, 4 females) to either 4 weeks of multi-strain probiotic (PRO) or visually identical placebo (PLB). (**B**) Dietary control 24 h pre-Marathon and food diary recording by RFPM. (**C**). Race day protocol commenced with blood and biopsy sampling at 07:00, followed by a controlled breakfast. Track Marathon commenced at 12:00, immediately following which blood and biopsy samples were collected again. Samples were immediately processed at the on-site laboratory and frozen stored at −80 °C. (**D**) Sample preparation and analysis by ^1^H-NMR. Peaks were assigned using Chenomx. Full parameters sets are deposited along with raw and processed spectra in the EMBL-EBI repository MetaboLights. (**E**) Univariate and multivariate data analysis were performed in the statistical software R, and pathway elucidation was undertaken in MetaboAnalyst and MetExplorer. Figure created with BioRender.com.

During the 24 h before the Marathon race, participants consumed a standardised high CHO, low fibre diet (per kg body mass: 8.0 g CHO (0.28 g fibre); 2.0 g protein; 1.0 g fat). Compliance with the diet was confirmed with food diaries and the remote food photography method [15]. After an overnight fast, participants reported to the laboratory at ~ 07:00 h and resting venous blood samples and muscle (vastus lateralis) biopsies were taken. Participants were then provided with a standardised breakfast (572 kcal; 128 g CHO (4.4 g fibre), 7 g protein, 3.5 g fat, and a minimum of 500 mL water) before a pre-race venous blood sample was collected. Participants performed self-selected warmups before a race briefing to reiterate in-race nutrition. The race started at 12:00 pm. Runners ran the 42,195 m race on a synthetic 400 m outdoor track (105.48 laps), which was in close proximity to the laboratory. Participants were fed CHO gels at a rate of 66 g/h during the Marathon race. Weather conditions throughout the race were as follows: temperature: 16–17 °C; wind speed: 8–16 km/h^−1^; precipitation: 0 mm. Immediately post-Marathon, blood samples and muscle biopsies were taken in the same manner as pre-Marathon. A schematic overview of the study design is presented in Figure 1.

### 2.1. Effects of Multi-Strain Probiotic Supplementation on the Alterations to the Serum Metabolome Induced by Marathon Running

**Figure 2:**
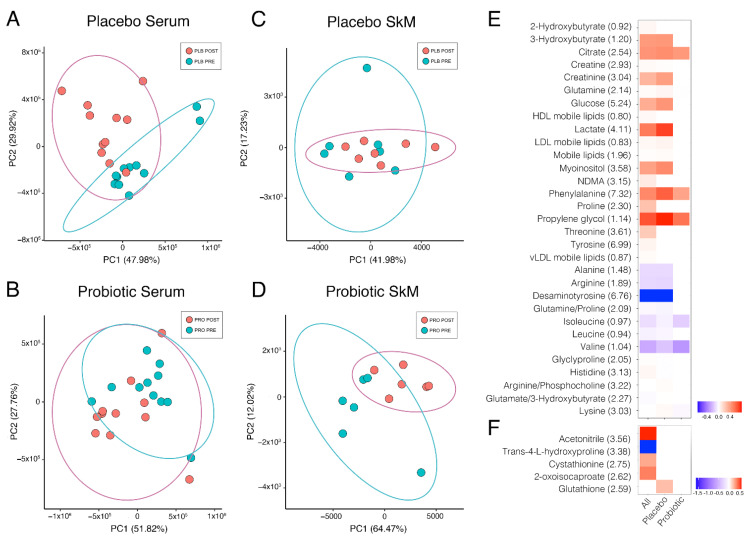
Pre- and post-race comparison via principal component analysis of serum placebo group (**A**), serum probiotic group (**B**), skeletal muscle placebo group (**C**), and skeletal muscle probiotic group (**D**). For clarity, ellipses show the 95% confidence region. Heatmap of metabolites significantly different pre- and post-Marathon for (**E**) serum and skeletal muscle tissue (**F**). Fold change calculated as a ratio of the mean for each listed metabolite peak pre-Marathon to post-Marathon. Data presented as binary logarithm (log2) to indicate whether each metabolite level has increased (greater than 0, red) or decreased (less than 0, blue) between sampling points. Not all participants were willing to provide a muscle biopsy, therefore PLB *n* = 7, PRO *n* = 6 for skeletal muscle samples. Values in brackets are the position of the metabolite peak in the NMR spectrum in ppm.

Unsupervised multivariate analysis of the serum groups by principal component analysis (PCA) was performed to determine if any underlying structure was present in the data (Figure 2C,D), and we observed that post-Marathon, a greater difference was observed in the PLB group than in PRO. Within-group differences pre- and post-Marathon were then explored by univariate tests for all serum buckets, which identified 94 significant buckets of which there were 36 different identities and 17 were non-overlapped metabolites in placebo. In contrast, the probiotic group identified only 15 buckets significantly different, of which there were seven different identities (all non-overlapped metabolites). Metabolites involved in energy metabolism predominated the significantly altered metabolite dataset; 2-hydorxybutryate increased in both groups and is an early marker for both insulin resistance and impaired glucose regulation that appears due to increased lipid oxidation and oxidative stress, and notably can increase glutathione synthesis. Glutathione was elevated in skeletal muscle biopsy samples, but only in PLB in our dataset. We also detected a significant increase in 3-hydroxybutyrate, which is metabolised by 3-hydroxybutyrate dehydrogenase to form acetoacetate, using NAD^+^ as an electron acceptor. The concentration of 3-hydroxybutyrate in blood is elevated in ketosis and was also only elevated in the PLB condition highlighting a potential switch to fatty acid metabolism over carbohydrate metabolism in this group. Glucose and tricarboxylic acid cycle intermediates such as citrate and lactate were also significantly altered by Marathon running, predominantly in the PLB group. A number of amino acids were also significantly altered by Marathon running, including phenylalanine, proline, threonine, tyrosine, alanine, arginine, glutamine, the branched-chain amino acids (leucine, isoleucine and valine) histidine and lysine. Notably, some glucogenic amino acids and precursors, particularly alanine and arginine showed significant decreases following the Marathon only in PLB, pointing towards increased reliance on amino acids as a source of glucose production, especially considering the elevation of ketone bodies in the PLB group post-Marathon. A heat map of the significantly altered metabolites is presented in Figure 2, and the metabolite sets to which they map are listed in Table 1. The most overrepresented pathways, as determined by hypergeometric testing, identified aminoacyl t-RNA biosynthesis and amino acid synthesis, likely due to the presence of numerous amino acids predominating the list of significantly altered metabolites.

**Figure 3:**
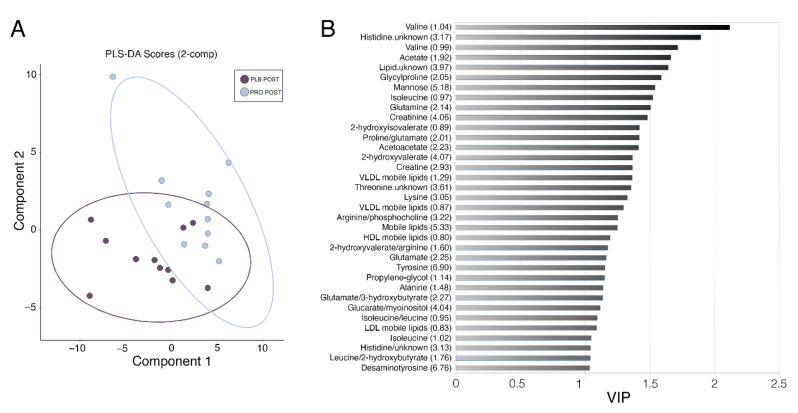
Partial least squares discriminant analysis (PLS-DA) of serum samples. (**A**) PLS-DA score plot representing component 1 and 2 for serum post-Marathon discriminating placebo (dark purple) vs. probiotic (light blue). Ellipses show the 95% confidence region. (**B**) VIP variables are presented for component 2 showed the most influential metabolites in the model. Metabolites with values over 1 were considered relevant. Values in brackets are the position of the metabolite peak in the NMR spectrum in ppm.

To attribute the differences between PRO and PLB group post-Marathon to particular metabolites, we implemented a multivariate approach by building a PLS-DA model of two components. This approach revealed combinations of metabolites that were able to discriminate between the two experimental groups post-Marathon (Figure 3A) with a ROC score of 0.83. Amino acids predominated as the most important variables of importance in projection (VIP; Figure 3B), with lipids, ketone bodies, creatinine, creatine, mannose and desaminotyrosine also identified as important variables (VIP score > 1). Many of these metabolites corroborated the findings from our univariate tests presented in Figure 2. Enriched metabolite sets reflected the predominance of amino acids in the model. Those metabolite sets with a Holm-adjusted *p*-value < 0.05 included aminoacyl-tRNA biosynthesis, d-glutamine and d-glutamate metabolism, and valine, leucine and isoleucine biosynthesis (Table 2).

### 2.2. Effects of Marathon Running and Probiotic Supplementation on the Skeletal Muscle Metabolome

Unsupervised multivariate analysis of the skeletal muscle biopsy groups by PCA showed no discernible difference post-Marathon between treatment groups (Figure 2C,D). Univariate analysis found only nine significant bins corresponding to five different identities amongst biopsy metabolite extracts between pre- and post-Marathon, and only one metabolite bucket annotated as glutathione was significantly different between PLB tissue extracts and none between PRO groups (Figure 2F).

## 3. Discussion

In order to generate new hypotheses and subsequently better characterise the mechanisms by which probiotic supplementation may incur beneficial physiological effects during Marathon running, the current study examined the effects of multi-strain probiotic supplementation in runners by serum metabolomics before and after a Marathon race. Principal component analysis (PCA) identified a greater difference in the post-Marathon serum metabolome in the PLB group vs. PRO. Univariate tests identified 17 non-overlapped metabolites in PLB, whereas only seven were identified in PRO. By building a PLS-DA model of two components, we revealed combinations of metabolites able to discriminate between PLB and PRO post-Marathon. PCA of muscle biopsies demonstrated no discernible difference post-Marathon between treatment groups. Collectively, the analysis shows for the first time that four weeks of multi-strain probiotic supplementation can attenuate the substantial change in the metabolome induced by running a Marathon and opens avenues for prospective research studies.

We recently reported novel findings that supplementation with a probiotic for 28-days prior to a track Marathon race was associated with lower incidence and severity of GI symptoms when compared with a placebo control and is associated with a better maintenance of running pace at the latter stages of the Marathon [10]. In this current study, the use of untargeted serum metabolomics on samples derived from the aforementioned track Marathon and by univariate analysis demonstrated that whilst 94 spectral buckets were altered in the placebo group in pre–post Marathon comparisons, only 15 were found to be significantly different in the probiotic cohort. Multivariate analysis highlighted that the difference between groups post-Marathon was mainly attributed to amino acids with lipids, ketone bodies, creatinine, creatine, mannose and desaminotyrosine also identified as important variables (Figure 3B). Together these data suggest a possible protection effect against the alteration of the pre-exercise metabolome derived from the probiotic supplement.

Our main theory is that probiotics offer a protective effect to the gut, preventing the substantial alteration of the serum amino acid profile post-Marathon. Endurance exercise and high-intensity interval exercise both cause gut damage and GI distress, presumably due to splanchnic hypoxia, oxidative stress, hyperthermia and mechanical stress related to exercise and malabsorption of CHO consumed before and during exercise [3,4,16]. Mounting evidence suggests that probiotics confer protective effects to the gut [7,9]. These protective effects are thought to be mediated by the release of molecules by the probiotic, activating signalling pathways responsible for the strengthening of tight junctions (intercellular adhesion complexes in epithelial and endothelial cells that control permeability), and may also confer cytoprotective properties by preventing disruption of tight junctions by damaging stimuli [reviewed by 16]). In support of this finding, we observed a significant decrease in the gut-derived microbial metabolite desaminotyrosine (DAT) in our placebo group after Marathon running, recently demonstrated to be a crucial anti-inflammatory molecule that modulates local and systemic immune homeostasis and protects gut barrier integrity [17]. It is also possible to speculate that the significant elevation of citrate in PLB could indicate inflammation since citrate is produced by immune cells and acts in a pro-inflammatory fashion [18]. The cause of this inflammatory response may be exercise-induced GI distress, which disrupts tight junctions and permits the release of lipopolysaccharide, in turn, act upon monocyte and macrophage activity and stimulating the release of pro-inflammatory cytokines [19]. Probiotics may also directly reduce the susceptibility of tight junctions to injurious stimuli. The scaffold protein zonula occludens (ZO)-1 and transmembrane protein occludin have been found to be significantly increased in the vicinity of the tight-junction structures in vivo following administration of *Lactobacillus plantarum* [20]. Another lactobacillus species, *Lactobacillus rhamnosus GG*, also confers protection of the gut to hydrogen peroxide-induced disruption of tight junctions and barrier function in Caco-2 cell monolayers [21]. Protein kinase C, extracellular related kinase 1/2 and mitogen activated protein kinase all appear to be key signalling proteins involved in the protective effects of probiotics on cells of the intestinal barrier [16]. Whilst a direct relationship between probiotic supplementation and gut integrity were not demonstrated by our dataset, it can be hypothesised that the attenuation of the substantial change in the metabolome induced by the injurious stimulus of Marathon running in our probiotic group, is due to the protective effects of probiotics on the structural properties of the gut. This may have important consequences for the absorption of nutrients during exercise, particularly CHO.

We hypothesise that probiotics can maintain intestinal integrity and therefore maintain CHO absorption and oxidation during prolonged exercise. Given that CHO ingestion may attenuate skeletal muscle degradation caused by exercise [22], maintenance of CHO absorption and utilisation during exercise may protect skeletal muscle during exercise and explain some of the alterations observed in the amino acid profile observed in the placebo group of this study. We report a greater decrease in the glucogenic amino acids alanine and arginine and a greater increase in 3-hydroxybutyrate in the placebo group, which point towards a shift to lipid metabolism and increased reliance on amino acids as a source of glucose production potentially via the glucose-alanine cycle. Previous reports [23,24], including a recent publication from our group [25], showed that CHO ingestion during prolonged endurance exercise (2 h at 55% W_max_) reduces endogenous glucose production by the liver to maintain blood glucose concentrations. It is important to consider that metabolomics measures metabolite abundances but not pathway activity, and therefore, future experiments could combine isotope tracers into metabolomic studies to determine pathway flux [26].

It is likely that the turnover of skeletal muscle also contributes to the change in the serum amino acid profile. Skeletal muscle protein turnover is increased following different modalities and durations of acute [27,28,29] and chronic endurance exercise [30,31]. Significant elevations in creatinine seen in this study may also indicate damage to skeletal muscle, which is common following Marathon running and long endurance events [32,33,34,35,36]. Increased protein turnover is exacerbated by low muscle CHO availability [22] and may be more pronounced when there is a higher mechanical load (acceleration/deceleration forces associated with running, for example). Notably, some glucogenic amino acids and precursors, particularly alanine and arginine, showed significant decreases following the Marathon only in the placebo group, despite regular CHO feeding during the Marathon in the form of CHO gels at a rate of 66 g/h. It can be suggested that there is an increased requirement for amino acids as a gluconeogenic substrate for the glucose-alanine cycle, potentially due to lower rates of CHO oxidation in the muscle. This hypothesis is supported by our recent findings that 4 weeks of multi-strain probiotic supplementation increased peak oxidation rates of ingested maltodextrin and total carbohydrate oxidation, accompanied by a reduction in fat oxidation whilst exercising for 2 h at 55% maximal aerobic power output [25]. A practical consequence of the increased protein turnover and amino acid oxidation due to endurance exercise is an elevation of the estimated daily protein requirements of endurance athletes, recently determined by the indicator amino acid oxidation method during a 3-day simulated training study (total running volume of 35 km) [37]. This work supports recent recommendations that protein nutrition for the endurance athlete is an important consideration, particularly after competition. These data also suggest that CHO feeding before and during Marathon racing is integral to support high rates of CHO utilisation and to limit glucose production from alternative gluconeogenic sources, such as protein. Despite high CHO intake in the day preceding the race (8 g per kg body mass), the pre-race breakfast (128 g) and CHO gels at a rate of 66 g/h, evidence of a shift to a greater reliance on alternative substrates for glucose production was still observed.

We also investigated the muscle metabolome from biopsy samples obtained at the same time as serum samples. Interestingly, we observed little difference between probiotic and placebo groups in the number of spectral buckets altered by the Marathon. Only one metabolite bucket annotated as glutathione (GSH) was significantly elevated in placebo tissue extracts, whereas no differences were observed between probiotic group samples. The redox state of GSH is an indicator of oxidative stress, and oxidised GSH increases in response to an exercise stimulus [38]. The current study did not measure oxidised and reduced GSH, only total glutathione. Resting concentrations of GSH increase as an adaptation to endurance exercise training, presumably to deal with frequent production of reactive oxygen species [39]. A systematic review and meta-analysis revealed that probiotic supplements improve the antioxidant defence system, increase the abundance of antioxidant enzymes and improve resistance to oxidizing agents [40]. Alternatively, metabolites secreted by probiotics, such as acetate (short-chain fatty acid; SCFA), have been shown to directly impact mitochondrial metabolism [41]. SCFA act as ligands for free fatty acid receptors 2 and 3 (FFAR2, FFAR3) that regulate glucose and fatty acid metabolism [42]. SCFA also regulates SIRT1 activity [41], a NAD-dependent deacetylase that deacetylates peroxisome proliferator initiated receptor gamma and coactivator 1 alpha (PGC-1α), a key player in mitochondrial biogenesis [43]. Whilst the concentration of the SCFA acetate was not significantly affected by Marathon running, basal and post-Marathon serum acetate concentration was higher in the probiotic group in the current study and one of the top 5 VIP variables in our PLS-DA model able to discriminate between placebo and probiotic post-Marathon (Figure 3B). Although we only had access to a small sample of biopsies (PLB *n* = 7, PRO *n* = 6 for skeletal muscle samples), these results may indicate that probiotic supplementation confers protection to oxidative stress and/or improves mitochondrial function during strenuous exercise and is worthy of further investigation.

Metabolomics studies are very sensitive to study design, and as such, intrinsic participant variation (such as sex, age genetic composition, habitual diet) is a limitation on this modest cohort size. Metabolomics, unlike other omics, is still very much a developmental field presenting difficulties in the identification of metabolites in complex mixtures, such as serum [44]. Furthermore, NMR metabolomics, albeit consistent and robust [45,46], are an untargeted technique and, as such, reports primary metabolites only [47]. Moreover, the technique presents challenges when interpreting metabolite changes in the biological context [48]. The pathways available on KEGG are not exhaustive for metabolite mapping and an overrepresentation of diseases, such as cancer, which may skew pathway analysis. All these limitations emphasise the need for more nutrition and exercise metabolomic datasets in order to improve biological contextualisation of exercise/nutrition metabolomics studies.

In conclusion, we present here a novel NMR metabolomics dataset in Marathon runners that received a probiotic or placebo supplement for four weeks prior to a track Marathon. Our data demonstrated a potentially protective effect of probiotic supplementation on the metabolic perturbations induced by a Marathon and raise new questions regarding the effects of probiotic supplements for exercise performance and metabolism. More sport and exercise nutrition studies are needed to improve the biological contextualisation of metabolomics data and to identify the mechanisms underpinning favourable metabolic effects of probiotics during endurance exercise. Moreover, it remains to be determined how the metabolic disturbance caused by the Marathon is resolved by time, and future studies should obtain multiple samples in the hours and days following the Marathon. From a practical perspective, the data provided here and in our previous reports [10,25] highlight the potential role probiotic bacteria may play in athlete metabolism and GI function and symptomology. Dose-response studies, investigation into different formulations of probiotic strains, and on the minimum effective duration of supplementation are still lacking.

## 4. Materials and Methods

### 4.1. Participants

Twenty-four runners (20 male, 4 female) participated in the study (Table 3). All participants were required to have run a Marathon race quicker than 5 h within the previous 2 years. Participant characteristics are presented in Table 3. All participants were free of medications, such as non-steroidal anti-inflammatory drugs (NSAIDs), antidepressants, or diuretics, nutritional supplements at the time of volunteering for the study, and any history of GI-related medical issues (IBS or abdominal surgery). After explaining the nature and risks of the experimental procedures to the participants, participants informed written consent was obtained. The study was approved by the University’s local ethics committee.

### 4.2. Experimental Design

In a double-blind, block randomised and matched-pairs design, participants underwent a 28-day period of supplementation consuming either a commercially available probiotic (PRO) or a visually identical placebo (PLB). Participants also consumed an additional supplement capsule on the morning of the race, two hours before the start. Participants were matched according to their most recent Marathon performance (PRO: 222 ± 46 min; PLB 220 ± 40 min) and body mass (Table 3). The probiotic supplement capsules contained the active strains *Lactobacillus acidophilus* CUL60, *Lactobacillus acidophilus* CUL21, *Bifidobacterium bifidum* CUL20 and *Bifidobacterium animalis* subs p. *Lactis* CUL34 (Proven Probiotics Ltd., Port Talbot, Wales, UK). The minimum dose was 25 billion colony forming units (CFU) per capsule. The placebo capsules were visually identical and consisted of 300 mg maltodextrin (Proven Probiotics Ltd., Port Talbot, Wales, UK). Participants were instructed to swallow the capsule daily after their first meal. The randomisation code was held by a third party, unlocked for analyses upon sample analysis completion. During the supplementation period, participants were instructed to refrain from all probiotic foods (i.e., fermented yogurts) and avoid any probiotic supplements.

### 4.3. Serum Sample Collection

Venous blood samples were collected in 8 mL serum separator vacutainers (BD), inverted 5–6 times and clotting proceeded at room temperature for 30 min prior to centrifugation (1300× *g*, 10 min, 4 °C). Serum was aliquoted and stored at −80 °C until analysis. All blood samples (pre- and post-Marathon) were processed in the same manner (with time between blood draw and freeze consistent throughout).

### 4.4. Tissue Sample Collection and Extraction

Muscle biopsies were extracted from the vastus lateralis pre- and immediately post-Marathon via perpendicular punch using 12-gauge × 10 cm biopsy gun needle (Bard Ltd., Crawley, UK) according to standard protocols [49]. Biopsies were transferred to sterile labelled cryovial tubes and were immediately snap-frozen in liquid N_2_ and stored at −80 °C until analysis. Tissue was resuspended in 50:50 *v/v* ice-cold acetonitrile:water (HPLC grade) and sonicated in 3 × 30 s bursts using a micro-tip sonicator (50 kHz) in an ice-bath. Extracts were then centrifuged at 4 °C 21,500× *g* for 5 min to pellet cell-debris with clarified supernatant lyophylised and stored at −80 °C prior to NMR acquisition. Muscle tissue samples were only collected from 13 participants (PLB *n* = 7, PRO *n* = 6) due to willingness to provide a biopsy.

### 4.5. Sample Preparation for NMR

Serum samples were prepared according to standard protocols [49] with NMR samples consisting of 50% serum, 10% ^2^H_2_O with 100 mM sodium phosphate buffer pH 7.4 and 0.1% azide. Lyophilised tissue extracts were resuspended to a final sample composition of 100% ^2^H_2_O with 100 mM sodium phosphate buffer pH 7.4, 100 μM Trimethylsiylpropionate (TSP) and 0.1% azide.

### 4.6. NMR Set-Up and Acquisition

Spectra were acquired on Bruker 700 MHz avance IIIHD spectrometer equipped with TCI cryoprobe and chilled autosampler (SampleJet, Ettlingen, Germany). Standard vendor pulse sequences were applied to collect 1D ^1^H-NMR spectra (cpmg1dpr). A Carr–Purcell–Meiboom–Gill (CPMG) edited pulse sequence was employed to attenuate signals from macromolecules present (proteins, etc.). Serum spectra were collected at 37 °C with 32 transients, whereas tissue extract spectra were collected at 25 °C with 128 transients for optimal sensitivity, with all other parameters kept constant. Full parameters sets were deposited along with raw and processed spectra in the EMBL European Bioinformatics Institute (EBI) repository MetaboLights with ID MTBLS1357 [50].

### 4.7. Spectra Processing and Quality Control

All spectra were automatically pre-processed at spectrometer by Fourier-transformation, phase correction and baseline correction using standard vendor routines (apk0.noe) and referenced either directly to TSP (tissue extracts) or indirectly via anomeric glucose signal (serum). Spectra were subjected to quality control criteria as recommended by Metabolomics Standards Initiative (MSI) [51,52]. Quality control criteria consisted of appraisal of baseline, line-width, residual water signal width, phase and signal-to-noise. Spectra were bucketed according to peaks boundaries defined with each bucket, the sum of the integral for that region divided by the region width.

### 4.8. Metabolite Annotation and Identification

Metabolites were annotated via the use of metabolite recognition software Chenomx (Chenomx v 8.2, Chenomx Ltd., Edmonton, AB, Canada), and the respective buckets were annotated prior to statistical analysis. Metabolites identities were confirmed (where possible) via comparison to the in-house metabolite library.

### 4.9. Statistical Analysis

Spectra were normalised via the probablistic quotient normalisation (PQN) method [53]. Samples of participants before and after the Marathon were compared via paired Welch tests, in which *p*-values were corrected for false discovery rate by the Benjamini-Hochberg method, and an adjusted *p*-value of <0.05 was considered significant. Differences between placebo and probiotic samples were appraised with both univariate (Welch tests) and multivariate approaches, including principal component analysis (PCA) and partial least squares discriminant analysis (PLS-DA). All statistical analyses were performed with the statistical software R [54]. Multivariate models were applied, combining the metabolomics data (using one signal per metabolite) and the biochemistry variables measured in the participants. PLS-DA models were built using the package mixOmics [55]. The number of components to retain for each model was calculated via 50 times 5-fold cross-validation using seventy percent of the data (training data), using the function perf within mixOmics. The 30% data left was used to test the accuracy of the models (test data). Details of each model are presented in the results.

Significant metabolites after the univariate tests and most important variables from the PLS-DA model were used for metabolite set enrichment analysis independently. We used a combined approach: MetaboAnalyst 3.0 [56] was used to enquire about the enrichment of the default pathways sets and diseases sets; with MetExplore [57], we tested the significance for pathways within the database KEGG (release 92) for *Homo sapiens*.

## Figures and Tables

**Table 1:** Results of the overrepresentation analysis using the hypergeometric test to evaluate whether a particular metabolite set was represented more than expected by chance within the metabolites identified from our univariate analysis. One-tailed *p*-values are provided after adjusting for multiple testing.

Biological Process	Total	Expected	Hits	Raw *p*-Value	Holm Adjusted *p*-Value	FDR
Aminoacyl-tRNA biosynthesis	48	0.778	12	7.53 × 10^−13^	6.32 × 10^−11^	6.32 × 10^−11^
Valine, leucine and isoleucine biosynthesis	8	0.13	4	3.61 × 10^−6^	3.00 × 10^−4^	0.000152
Phenylalanine, tyrosine and tryptophan biosynthesis	4	0.0649	2	0.00149	0.122	0.0416
Phenylalanine metabolism	10	0.162	2	0.0105	0.85	0.22
Arginine biosynthesis	14	0.227	2	0.0204	1	0.301
Arginine and proline metabolism	38	0.616	3	0.0218	1	0.301
Valine, leucine and isoleucine degradation	40	0.649	3	0.025	1	0.301
Neomycin, kanamycin and gentamicin biosynthesis	2	0.0324	1	0.0322	1	0.338
Galactose metabolism	27	0.438	2	0.0692	1	0.62

**Table 2:** Results of the overrepresentation analysis using the hypergeometric test to evaluate whether a particular metabolite set was represented more than expected by chance within the metabolites identified from PLS-DA analysis (VIP scores > 1). One-tailed *p*-values are provided after adjusting for multiple testing.

Biological Process	Total	Expected	Hits	Raw *p*-Value	Holm Adjusted *p*-Value	FDR
Aminoacyl-tRNA biosynthesis	48	0.725	10	3.10 × 10^−10^	2.61 × 10^−8^	2.61 × 10^−8^
d-Glutamine and d-glutamate metabolism	6	0.0907	4	5.85 × 10^−7^	4.85 × 10^−5^	2.46 × 10^−5^
Valine, leucine and isoleucine biosynthesis	8	0.121	3	0.000161	0.0132	0.00451
Arginine biosynthesis	14	0.212	3	0.000986	0.0798	0.0207
Valine, leucine and isoleucine degradation	40	0.604	4	0.00253	0.202	0.0425
Nitrogen metabolism	6	0.0907	2	0.00316	0.25	0.0442
Ascorbate and aldarate metabolism	8	0.121	2	0.00579	0.452	0.0695
Alanine, aspartate and glutamate metabolism	28	0.423	3	0.00772	0.595	0.0811
Glyoxylate and dicarboxylate metabolism	32	0.484	3	0.0112	0.854	0.105
Arginine and proline metabolism	38	0.574	3	0.018	1	0.151
Butanoate metabolism	15	0.227	2	0.0204	1	0.155
Histidine metabolism	16	0.242	2	0.0231	1	0.161

**Table 3:** Participant characteristics. Values are means ± SD. Differences between groups for all measures were not significant (*p* < 0.05). LT = lactate threshold. Gender split (10 Males and 2 Females per group. Values are running speed at LT. PLB = placebo, PRO = probiotic.

	PLB		PRO	
	Mean ± SD	Range	Mean ± SD	Range
Age (years) Height (m) Body Mass (kg) VO_2peak_ (mL·kg·min^−1^) LT (km·h^−1^) Most recent Marathon time (min)	36.1 ± 7.5 1.75 ± 1.11 73.5 ± 11.3 56.4 ± 8.6 11.9 ± 1.9 220 ± 40	29–50 1.52–1.86 48–95 47.2–70.0 9–16.0 150–283	34.8 ± 6.9 1.79 ± 0.6 76.5 ± 9.4 57.6 ± 8.0 12.3 ± 1.9 222 ± 46	22–43 1.68–1.90 61–92 48.1–66.7 10–15.5 152–315

## Data Availability

Full parameters sets are deposited along with raw and processed spectra in the EMBL European Bioinformatics Institute (EBI) repository MetaboLights with ID MTBLS1357 [50].

## References

[1] Pugh J.N., Kirk B., Fearn R., Morton J.P., Close G.L. (2018). Prevalence, Severity and Potential Nutritional Causes of Gastrointestinal Symptoms during a Marathon in Recreational Runners. Nutrients.

[2] Keeffe E.B., Lowe D.K., Goss J.R., Wayne R. (1984). Gastrointestinal symptoms of marathon runners. West J. Med..

[3] De Oliveira E.P., Burini R.C. (2014). Carbohydrate-dependent, exercise-induced gastrointestinal distress. Nutrients.

[4] Van Wijck K., Lenaerts K., van Loon L.J., Peters W.H., Buurman W.A., Dejong C.H. (2011). Exercise-induced splanchnic hypoperfusion results in gut dysfunction in healthy men. PLoS ONE.

[5] Currell K., Jeukendrup A.E. (2008). Superior endurance performance with ingestion of multiple transportable carbohydrates. Med. Sci. Sports Exerc..

[6] Coyle E.F., Hagberg J.M., Hurley B.F., Martin W.H., Ehsani A.A., Holloszy J.O. (1983). Carbohydrate feeding during prolonged strenuous exercise can delay fatigue. J. Appl. Physiol. Respir. Environ. Exerc. Physiol..

[7] Valdes A.M., Walter J., Segal E., Spector T.D. (2018). Role of the gut microbiota in nutrition and health. BMJ.

[8] Wosinska L., Cotter P.D., O’Sullivan O., Guinane C. (2019). The Potential Impact of Probiotics on the Gut Microbiome of Athletes. Nutrients.

[9] Jager R., Mohr A.E., Carpenter K.C., Kerksick C.M., Purpura M., Moussa A., Townsend J.R., Lamprecht M., West N.P., Black K. (2019). International Society of Sports Nutrition Position Stand: Probiotics. J. Int. Soc. Sports Nutr..

[10] Pugh J.N., Sparks A.S., Doran D.A., Fleming S.C., Langan-Evans C., Kirk B., Fearn R., Morton J.P., Close G.L. (2019). Four weeks of probiotic supplementation reduces GI symptoms during a marathon race. Eur. J. Appl. Physiol..

[11] Kekkonen R.A., Vasankari T.J., Vuorimaa T., Haahtela T., Julkunen I., Korpela R. (2007). The effect of probiotics on respiratory infections and gastrointestinal symptoms during training in marathon runners. Int. J. Sport Nutr. Exerc. Metab..

[12] Roberts J.D., Suckling C.A., Peedle G.Y., Murphy J.A., Dawkins T.G., Roberts M.G. (2016). An Exploratory Investigation of Endotoxin Levels in Novice Long Distance Triathletes, and the Effects of a Multi-Strain Probiotic/Prebiotic, Antioxidant Intervention. Nutrients.

[13] Wikoff W.R., Anfora A.T., Liu J., Schultz P.G., Lesley S.A., Peters E.C., Siuzdak G. (2009). Metabolomics analysis reveals large effects of gut microflora on mammalian blood metabolites. Proc. Natl. Acad. Sci. USA.

[14] Stander Z., Luies L., Mienie L.J., Keane K.M., Howatson G., Clifford T., Stevenson E.J., Loots D.T. (2018). The altered human serum metabolome induced by a marathon. Metabolomics.

[15] Martin C.K., Correa J.B., Han H., Allen H.R., Rood J.C., Champagne C.M., Gunturk B.K., Bray G.A. (2012). Validity of the Remote Food Photography Method (RFPM) for estimating energy and nutrient intake in near real-time. Obesity.

[16] Rao R.K., Samak G. (2013). Protection and Restitution of Gut Barrier by Probiotics: Nutritional and Clinical Implications. Curr. Nutr. Food Sci..

[17] Wei Y., Gao J., Kou Y., Liu M., Meng L., Zheng X., Xu S., Liang M., Sun H., Liu Z. (2020). The intestinal microbial metabolite desaminotyrosine is an anti-inflammatory molecule that modulates local and systemic immune homeostasis. FASEB J..

[18] Palsson-McDermott E.M., O’Neill L.A.J. (2020). Targeting immunometabolism as an anti-inflammatory strategy. Cell Res..

[19] Dokladny K., Zuhl M.N., Moseley P.L. (2016). Intestinal epithelial barrier function and tight junction proteins with heat and exercise. J. Appl. Physiol..

[20] Karczewski J., Troost F.J., Konings I., Dekker J., Kleerebezem M., Brummer R.J., Wells J.M. (2010). Regulation of human epithelial tight junction proteins by Lactobacillus plantarum in vivo and protective effects on the epithelial barrier. Am. J. Physiol. Gastrointest. Liver Physiol..

[21] Seth A., Yan F., Polk D.B., Rao R.K. (2008). Probiotics ameliorate the hydrogen peroxide-induced epithelial barrier disruption by a PKC- and MAP kinase-dependent mechanism. Am. J. Physiol. Gastrointest. Liver Physiol..

[22] Borsheim E., Cree M.G., Tipton K.D., Elliott T.A., Aarsland A., Wolfe R.R. (2004). Effect of carbohydrate intake on net muscle protein synthesis during recovery from resistance exercise. J. Appl. Physiol..

[23] Coyle E.F., Jeukendrup A.E., Wagenmakers A.J., Saris W.H. (1997). Fatty acid oxidation is directly regulated by carbohydrate metabolism during exercise. Am. J. Physiol..

[24] Jeukendrup A.E., Moseley L. (2010). Multiple transportable carbohydrates enhance gastric emptying and fluid delivery. Scand. J. Med. Sci. Sports.

[25] Pugh J.N., Wagenmakers A.J.M., Doran D.A., Fleming S.C., Fielding B.A., Morton J.P., Close G.L. (2020). Probiotic supplementation increases carbohydrate metabolism in trained male cyclists: A randomized, double-blind, placebo-controlled crossover trial. Am. J. Physiol. Endocrinol. Metab..

[26] Jang C., Chen L., Rabinowitz J.D. (2018). Metabolomics and Isotope Tracing. Cell.

[27] Carraro F., Hartl W.H., Stuart C.A., Layman D.K., Jahoor F., Wolfe R.R. (1990). Whole body and plasma protein synthesis in exercise and recovery in human subjects. Am. J. Physiol..

[28] Tipton K.D., Ferrando A.A., Williams B.D., Wolfe R.R. (1996). Muscle protein metabolism in female swimmers after a combination of resistance and endurance exercise. J. Appl. Physiol..

[29] Harber M.P., Konopka A.R., Douglass M.D., Minchev K., Kaminsky L.A., Trappe T.A., Trappe S. (2009). Aerobic exercise training improves whole muscle and single myofiber size and function in older women. Am. J. Physiol. Regul. Integr. Comp. Physiol..

[30] Short K.R., Vittone J.L., Bigelow M.L., Proctor D.N., Nair K.S. (2004). Age and aerobic exercise training effects on whole body and muscle protein metabolism. Am. J. Physiol. Endocrinol. Metab..

[31] Pikosky M.A., Gaine P.C., Martin W.F., Grabarz K.C., Ferrando A.A., Wolfe R.R., Rodriguez N.R. (2006). Aerobic exercise training increases skeletal muscle protein turnover in healthy adults at rest. J. Nutr..

[32] Weight L.M., Alexander D., Jacobs P. (1991). Strenuous exercise: Analogous to the acute-phase response?. Clin. Sci..

[33] Starkie R.L., Rolland J., Angus D.J., Anderson M.J., Febbraio M.A. (2001). Circulating monocytes are not the source of elevations in plasma IL-6 and TNF-alpha levels after prolonged running. Am. J. Physiol. Cell Physiol..

[34] Kratz A., Lewandrowski K.B., Siegel A.J., Chun K.Y., Flood J.G., Van Cott E.M., Lee-Lewandrowski E. (2002). Effect of marathon running on hematologic and biochemical laboratory parameters, including cardiac markers. Am. J. Clin. Pathol..

[35] Smith J.E., Garbutt G., Lopes P., Tunstall Pedoe D. (2004). Effects of prolonged strenuous exercise (marathon running) on biochemical and haematological markers used in the investigation of patients in the emergency department. Br. J. Sports Med..

[36] Suzuki K., Peake J., Nosaka K., Okutsu M., Abbiss C.R., Surriano R., Bishop D., Quod M.J., Lee H., Martin D.T. (2006). Changes in markers of muscle damage, inflammation and HSP70 after an Ironman Triathlon race. Eur. J. Appl. Physiol..

[37] Kato H., Suzuki K., Bannai M., Moore D.R. (2016). Protein Requirements Are Elevated in Endurance Athletes after Exercise as Determined by the Indicator Amino Acid Oxidation Method. PLoS ONE.

[38] Sen C.K., Atalay M., Hanninen O. (1994). Exercise-induced oxidative stress: Glutathione supplementation and deficiency. J. Appl. Physiol..

[39] Leeuwenburgh C., Hollander J., Leichtweis S., Griffiths M., Gore M., Ji L.L. (1997). Adaptations of glutathione antioxidant system to endurance training are tissue and muscle fiber specific. Am. J. Physiol..

[40] Heshmati J., Farsi F., Shokri F., Rezaeinejad M., Almasi-Hashiani A., Vesali S., Sepidarkish M. (2018). A systematic review and meta-analysis of the probiotics and synbiotics effects on oxidative stress. J. Funct. Foods.

[41] Clark A., Mach N. (2017). The Crosstalk between the Gut Microbiota and Mitochondria during Exercise. Front. Physiol..

[42] Den Besten G., van Eunen K., Groen A.K., Venema K., Reijngoud D.J., Bakker B.M. (2013). The role of short-chain fatty acids in the interplay between diet, gut microbiota, and host energy metabolism. J. Lipid Res..

[43] Rodgers J.T., Lerin C., Haas W., Gygi S.P., Spiegelman B.M., Puigserver P. (2005). Nutrient control of glucose homeostasis through a complex of PGC-1alpha and SIRT1. Nature.

[44] Wishart D.S. (2011). Advances in metabolite identification. Bioanalysis.

[45] Dona A.C., Jimenez B., Schafer H., Humpfer E., Spraul M., Lewis M.R., Pearce J.T., Holmes E., Lindon J.C., Nicholson J.K. (2014). Precision high-throughput proton NMR spectroscopy of human urine, serum, and plasma for large-scale metabolic phenotyping. Anal. Chem..

[46] Jimenez B., Holmes E., Heude C., Tolson R.F., Harvey N., Lodge S.L., Chetwynd A.J., Cannet C., Fang F., Pearce J.T.M. (2018). Quantitative Lipoprotein Subclass and Low Molecular Weight Metabolite Analysis in Human Serum and Plasma by (1)H NMR Spectroscopy in a Multilaboratory Trial. Anal. Chem..

[47] Psychogios N., Hau D.D., Peng J., Guo A.C., Mandal R., Bouatra S., Sinelnikov I., Krishnamurthy R., Eisner R., Gautam B. (2011). The human serum metabolome. PLoS ONE.

[48] Viant M.R., Kurland I.J., Jones M.R., Dunn W.B. (2017). How close are we to complete annotation of metabolomes?. Curr. Opin. Chem. Biol..

[49] Beckonert O., Keun H.C., Ebbels T.M., Bundy J., Holmes E., Lindon J.C., Nicholson J.K. (2007). Metabolic profiling, metabolomic and metabonomic procedures for NMR spectroscopy of urine, plasma, serum and tissue extracts. Nat. Protoc..

[50] Haug K., Salek R.M., Conesa P., Hastings J., de Matos P., Rijnbeek M., Mahendraker T., Williams M., Neumann S., Rocca-Serra P. (2013). MetaboLights--an open-access general-purpose repository for metabolomics studies and associated meta-data. Nucleic Acids Res..

[51] Salek R.M., Steinbeck C., Viant M.R., Goodacre R., Dunn W.B. (2013). The role of reporting standards for metabolite annotation and identification in metabolomic studies. Gigascience.

[52] Sumner L.W., Amberg A., Barrett D., Beale M.H., Beger R., Daykin C.A., Fan T.W., Fiehn O., Goodacre R., Griffin J.L. (2007). Proposed minimum reporting standards for chemical analysis Chemical Analysis Working Group (CAWG) Metabolomics Standards Initiative (MSI). Metabolomics.

[53] Kohl S.M., Klein M.S., Hochrein J., Oefner P.J., Spang R., Gronwald W. (2012). State-of-the art data normalization methods improve NMR-based metabolomic analysis. Metabolomics.

[54] R Core Team R: A Language and Environment for Statistical Computing. https://www.R-project.org/.

[55] Le Cao K.-A., Rohart F., Gonzalez I., Dejean S. mixOmics: Omics Data Integration Project, R package version 6.1.3; 2017.

[56] Xia J., Sinelnikov I.V., Han B., Wishart D.S. (2015). MetaboAnalyst 3.0--making metabolomics more meaningful. Nucleic Acids Res..

[57] Cottret L., Frainay C., Chazalviel M., Cabanettes F., Gloaguen Y., Camenen E., Merlet B., Heux S., Portais J.C., Poupin N. (2018). MetExplore: Collaborative edition and exploration of metabolic networks. Nucleic Acids Res..

